# Consecutive 5-year outcomes of chorionic villus sampling at a tertiary center

**DOI:** 10.1097/MD.0000000000041582

**Published:** 2025-02-14

**Authors:** Melda Kuyucu, Kadri Murat Erdogan, Duygu Adiyaman, Bahar Konuralp Atakul, Hakan Golbasi, Yasar Bekir Kutbay, Ayse Filiz Gokmen Karasu, Mehmet Ozeren

**Affiliations:** a Department of Obstetrics and Gynecology, Bezmialem Vakif University Medical Faculty, Istanbul, Turkey; b Department of Obstetrics and Gynecology, Division of Perinatology, Tepecik Training and Research Hospital, Izmir, Turkey.; c Genetic Diagnosis Center, Izmir City Hospital, Izmir, Turkey; d Department of Obstetrics and Gynecology, Division of Perinatology, Ulm University, Ulm, Germany; e Department of Obstetrics and Gynecology, Division of Perinatology, Izmir City Hospital, Izmir, Turkey

**Keywords:** chorionic villus sampling, fetal structural abnormalities, first-trimester screen-positive test, noninvasive prenatal test, prenatal diagnostic invasive procedures

## Abstract

This study shares our 5-year experience with chorionic villus sampling (CVS) and analyzes the indications, results, and complications of this procedure. We conducted a retrospective analysis of data from singleton pregnancies that underwent CVS between 2015 and 2020 at the Maternal-Fetal Medicine Unit of Health Science University, Izmir Tepecik Research, and Training Hospital. Maternal demographics, indications, karyotype results, and pregnancy outcomes were recorded. We retrospectively analyzed data from 468 CVS procedures, conducted between 2015 and 2020. The most common indications for CVS were positive screening test results in the first trimester, fetal structural abnormalities, and increased nuchal translucency (NT) observed during ultrasound. Fetal structural abnormalities had the highest detection rate, at 34.5% for chromosomal abnormalities, followed by increased NT and first-trimester screen-positive test results (26.9% and 11.3%), respectively. The culture success rate was 96.3% (451 out of 468). The most prevalent chromosomal abnormalities were numerical, including Trisomy 21 (10.9%), Trisomy 18 (4.2%), and Trisomy 13 (1.9%). Results could not be obtained in 17 patients (3.6%); 12 (2.5%) were due to insufficient samples and culture failure, while 5 (1.06%) were due to maternal contamination. Amniocentesis was required as a secondary sampling in 24 cases (5.1%) and performed in 17 cases (3.6%). This study emphasizes the significance of CVS in prenatal diagnosis and the management of high-risk pregnancies. However, we must be aware of the associated risks and limitations, which include culture success rates, inconclusive results, and the occasional need for secondary sampling.

## 1. Introduction

Chromosomal abnormalities are more commonly observed in early pregnancy loss, with an estimated incidence of 1/150 in live births.^[1]^ The primary target of prenatal genetic screening is to be able to detect chromosomal abnormalities, especially Down syndrome, the most common viable autosomal aneuploidy among live-born infants.^[2]^ Pregnant women at an elevated risk of chromosomal abnormalities encompass those of advanced maternal age, history of pregnancy with a chromosomal abnormality, parental chromosomal translocation, positive test results on aneuploidy screening, and, fetal structural abnormalities on ultrasound examination.^[3]^ Despite cell-free DNA (cf-DNA) emerging as the most sensitive and specific screening test for common aneuploidies, it is not yet a definitive diagnostic method for chromosomal abnormalities. Therefore, decisions regarding pregnancy termination should not be solely based on cf-DNA test results.^[4]^ The parents should be informed about alternatives to prenatal diagnostic tests, the most common conventional methods, such as chorionic villus sampling (CVS), second-trimester amniocentesis (AC) and, fetal blood sampling or postnatal newborn evaluation, with postnatal genetic testing, those in cases where prenatal diagnosis is declined.

CVS is typically performed during the first trimester, between 10 and 13 + 6 weeks of gestation by transabdominal or transcervical approach. The most common complications associated with CVS include fetal loss, vaginal bleeding, infection and rupture of membranes.^[5]^ Despite certain technical challenges and a relatively higher rate of culture failure associated with CVS, its major advantages encompass expedited final results, relatively shorter waiting times, and decreased parental anxiety due to early implementation time.^[6]^ Moreover, in cases of chromosomal abnormality detection, there are no medical and ethical risks associated with late pregnancy termination in patients requesting termination.

Our aim in this study is to evaluate CVS outcomes and the indications, results, and complications associated with this procedure.

## 2. Material and methods

The data of singleton pregnancies who underwent CVS between 2015 and 2020 in the Maternal-Fetal Medicine Unit of Health Science University, Izmir Tepecik Research, and Training Hospital were analyzed retrospectively. The study protocol was approved by the local ethics committee (number 2021/5-19 and registration date 17/05/2021).

The study included cases undergoing CVS for genetic diagnosis, excluding cases involving AC and fetal blood sampling. The indications of prenatal invasive procedure were a positive screening test in the first -trimester (combined; the high-risk cutoff value was accepted as > 1/250), major fetal structural anomaly, increased nuchal translucency (NT), history of pregnancy complicated with fetal trisomy, advanced maternal age and maternal request, and parental chromosomal disorder carrier. Increased NT was defined as NT ≥ 3 mm in the first-trimester ultrasound. Advanced maternal age was defined as ≥ 35 years at delivery. All patients were informed about the benefits, potential complications, and limitations of CVS and alternatives (second-trimester AC, cf-DNA, or postnatal newborn evaluation with genetic testing) for genetic counseling. Written informed consent was obtained from all patients undergoing invasive diagnostic testing, and CVS was planned according to the gestational age.

Gestational age was calculated from the last menstrual date and confirmed by crown-rump length on first-trimester ultrasound. CVS was performed between 11 and 14 weeks of gestation via transabdominal access. Utilizing the double-needle technique under ultrasound guidance, an 18-gauge needle was inserted without local anesthesia to reach the placenta. After retracting the stylet and replacing it with a 20-gauge needle, the placental villi were aspirated manually by an assistant.^[3]^ The procedure was performed under aseptic conditions, under continuous ultrasound guidance, and without local anesthesia by a fellow assistant under supervision by a maternal-fetal specialist. All samples were sent to the genetics laboratory, where both quantitative fluorescence polymerase chain reaction and conventional karyotyping were performed. Fetal viability, position, and placental location were checked before procedures. The presence of fetal heart rate, amniotic fluid volume, and hemorrhage area were inspected after the procedure. Rh-negative and indirect Coombs-negative patients received 300 mg of Rh IGG to prevent Rh alloimmunization.

Maternal demographics (maternal age, gravidity, parity, refugee), week of gestation, pregnancy outcomes (termination, spontaneous miscarriage, procedure-related miscarriage, stillbirths, and live births), indications, and karyotype results (numerical and structural abnormalities) were recorded. Spontaneous miscarriage was defined as an abortus before 24 gestational weeks, and procedure-related miscarriage was defined as pregnancy losses with and without chromosomal abnormality after an invasive procedure within 15 days were obtained from medical records of the hospital database system.

Statistical analyses were performed as descriptive analysis and the Statistical Package for the Social Sciences version 26.0 (IBM Corporation, Armonk) was used for data analysis.

## 3. Results

**Figure 1.**
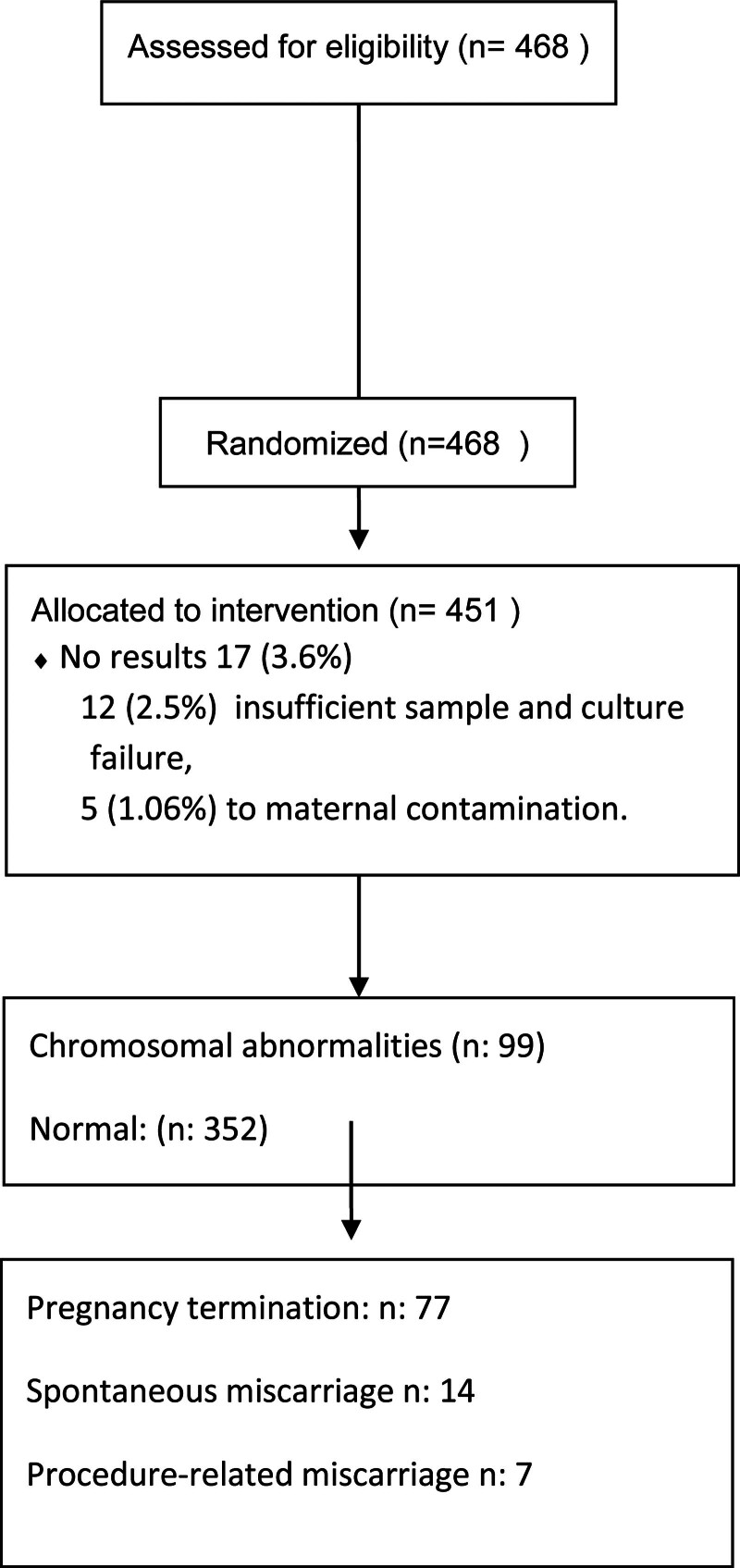
Study flow chart and outcomes of patients undergoing chorionic villus sampling (CVS). CVS = chorionic villus sampling.

In this present study, data from a total of 468 prenatal invasive diagnostic procedures, undergoing CVS, between 2015 and 2020 were retrospectively analyzed. The study flow chart and outcomes of patients undergoing CVS are shown in Figure 1. The median maternal age was found to be 32 (16–45) years, with a median gestational week at the time of the procedure was 13 (11–14). Among the procedures, 99 (21.1%) cases resulted in a diagnosis of chromosomal abnormalities. Maternal and general characteristics are shown in Table 1.

**Table 1:** Maternal demographics.

	CVS (n = 468)	
Maternal age	32 (16–45)	31.7 ± 6.4
Gravida	2 (1–7)	2.5 ± 1.2
Parity	1 (0–6)	1.1 ± 1.0
Gestational week	13 (11–14)	12.6 ± 0.95
Refugee (%)	12 (2.5%)	
Chromosomal abnormalities (%)	99 (21.1%)	

Data are given as median (min–max), mean ± SD, and n (%).

CVS = chorionic villus sampling.

Table 2 shows the indications and detection rates of chromosomal abnormalities. The most common indications for CVS were positive screening test results in the first trimester (combined), major fetal structural abnormalities, and increased NT observed on ultrasound. Fetal structural abnormalities had the highest detection rate, at 34.5% for chromosomal abnormality followed by increased NT, and first-trimester screen-positive test results (26.9% and 11.3%), respectively.

**Table 2:** Indications and detection rates of CVS procedures.

Indications	Total (n = 468)	Chromosomal abnormality (n = 99)	DR of chromosomal abnormality (%)
Screen positive test results (first-trimester)	185 (39.5%)	23 (23.2%)	12.4
Fetal structural anomaly (cystic hygroma, megacystis or nonimmune hydrops fetalis)	142 (30.3%)	49 (49.4%)	34.5
Increased NT	89 (19.0%)	24 (24.2%)	26.9
Advanced maternal age and maternal request	28 (5.9%)	2 (2.0%)	7.1
History of pregnancy with a chromosomal abnormality (fetal trisomy)	13 (2.7%)	1 (1.0%)	7.6
Parental chromosomal disorder carrier	11 (2.3%)	0	0
Total	468	99	21.1%

Data are given as n (%).

CVS = chorionic villus sampling, DR = detection rate, NT = nuchal translucency.

The most prevalent chromosomal abnormalities were numerical; including Trisomy 21 (10.9%), Trisomy 18 (4.2.%), and 13 (1.9%). The culture success rate was 96.3% (451/468). No results were obtained in 17 (3.6%) patients; 12 (2.5%) attributed to insufficient sample and culture failure, 5 (1.06%) to maternal contamination. AC was required as a secondary sampling for 24 cases (5.1%) and performed in 17 (3.6%) cases. True mosaicism was identified in 1 patient who underwent AC, while placental mosaicism could not be excluded in another mosaic case where AC was declined. Three AC results for maternal contamination revealed 2 normal and 1 trisomy 21. For cases with no results due to culture failure, subsequent AC results in 6 patients were normal. Other ACs were conducted upon laboratory request, with results consistent with CVS in 7 cases, all indicated normal. Parental karyotype analysis was conducted on 3 patients for the fetal structural chromosomal abnormalities. For the 2 fetal karyotypes, 46,.., rob (21,21)(q10;q10),+21 and 47,..,+mar, the presence of normal parental karyotypes led to the consideration that these chromosomal abnormalities might have arisen from de novo mutations or parental gonadal mosaicism. For the third; the maternal karyotype was identical to that of the fetus, both showing 45,.., rob (13;14)(q10;q10). Detailed chromosomal results and structural defects are shown in Tables 3 and 4.

**Table 3:** Results of chromosomal analysis.

	CVS (n = 468)	Termination rate of pregnancy (n %)
Normal	352 (75.2)	24 (6.8)
Total chromosomal abnormality	99 (21.1)	53 (53.5)
Numeric abnormalities	94 (20.0)	46 (48.9)
Trisomy 21	51 (10.9)	25 (49.0)
Trisomy 18	20 (4.2)	12 (60.0)
Trisomy 13	9 (1.9)	5 (55.5)
Monosomy X	9 (1.9)	3 (33.3)
Triploidy	3 (0.6)	0
Trisomy 20	1 (0.2)	0
Trisomy 1	1 (0.2)	1 (100)
Structural abnormalities	3 (0.6)	2 (66.6)
Mosaism	2 (0.42)	0
No results (culture failure/inadequate sample)	12 (2.5)	1 (8.3)
Maternal contamination	5 (1.06)	4 (80)

Data are given as n (%).

CVS = chorionic villus sampling.

**Table 4:** Other chromosomal abnormalities of CVS.

Mosaism	Structural abnormalities
45, X(12)/46,XY[30]	46,.., rob (21,21)(q10;q10),+21dn
45, X(10)/46,XY[26]	45,.., rob (13;14)(q10;q10) mat
	47,..,+mar

CVS = chorionic villus sampling, dn = de novo, mar = marker, mat = maternal, rob = Robertsonian.

A total of 77 (16.4%) patients opted for pregnancy termination, with 53 (68.8%) terminations related to chromosomal abnormalities and 24 (31.2%) due to fetal structural abnormalities incompatible with postnatal life. The spontaneous miscarriage rate was 2.9% and procedure-related miscarriage was 1.49%, with 0.64% of these fetuses having no chromosomal or structural abnormalities. Because our center is one of the largest perinatal referral centers in Turkey, most cases were followed up in their hospital and a total of 63 (13.2%) pregnancies were delivered in our center (61 live births, 2 stillbirths).

Only one patient experienced severe maternal complication following the CVS procedure- a uterine rupture in a woman with a previous cesarean section. The rupture was repaired by immediate operation.

## 4. Discussion

Chromosomal abnormalities, whether numerical or structural, have devastating effects on affected infants and their families. Accurate and early prenatal genetic diagnosis of both is crucial,^[1]^ with prenatal invasive diagnostic procedures serving as valuable tools in this regard, offering significant insights into fetal chromosomal abnormalities and aiding in informed decision-making for parents about the termination of pregnancy.^[7]^ In this study, we described our 5-year experience with CVS, a widely utilized procedure for diagnosing fetal chromosomal abnormalities, including distributions of indications, cytogenetic results, and complications.

The effectiveness of CVS as a prenatal diagnostic procedure is often assessed by its detection rate, extensively studied across various populations. Our study’s finding, with a 21.1% detection rate for chromosomal abnormalities, aligns with comparable rates reported in other similar studies (25.5% and 26.6).^[8,9]^ Fetal genetic abnormalities are a leading cause of first trimester miscarriages and CVS plays a critical role in the early detection of these genetic anomalies, thanks to its implementation at an early stage of pregnancy. However, it is important to note that detection rates using CVS may vary depending on the specific indications for the procedure. While positive screening test results in the firsttrimester, major fetal abnormalities, and increased NT detected by ultrasound examination showed common indications, the highest detection rate was associated with fetal abnormalities on ultrasound, increased NT and screen-positive test results, as in the study by Beksaç et al.^[10]^ The detection rate of each indication in our study population underscores the importance of ultrasound and early prenatal screening in identifying potential risks. The relationship between fetal structural malformations, increased NT, and chromosomal abnormalities, is well established.^[11,12]^ Advances in ultrasound imaging over the years, contributed to increased detection rates of fetal anomalies in early gestational weeks, emphasizing the importance of prenatal ultrasound examinations for early chromosomal anomaly diagnosis^[13]^ Up to fifty fetal structural malformations can be detected in the first trimester.^[14]^ Therefore prenatal ultrasound examination plays an important role early diagnosis of chromosomal abnormalities through this association. Not all fetuses with structural malformations have major chromosomal abnormalities^[15]^ because some malformations may be caused by environmental factors, single gene disorders, or unknown causes, and further genetic analysis (microarray analysis, CES, WES) may be required.^[16]^ Therefore, fetal structural malformations should prompt exhaustive consideration of chromosomal analysis to rule out chromosomal abnormalities as a cause. Notably, advanced maternal age and parental chromosomal disorder carriers had the lowest detection rates (7.4% and 0.0%). Invasive prenatal tests used to be done for advanced maternal age until recently, since ACOG stated in 2007 that advanced maternal age alone is not an indication for an invasive procedure.^[17]^ In our center, the noninvasive prenatal test was done for advanced maternal age^[18]^ and according to these results, noninvasive prenatal test appears to be an adequate alternative for these women to reduce unnecessary invasive interventions.

It is important to highlight that a subset of patients (3.6%) may require the performance of AC following CVS due to inconclusive results arising from factors such as insufficient sample material, culture failure, maternal contamination, or even to differentiate true fetal mosaicism from confined placental mosaicism. AC was required as a secondary sampling for 24 cases (5.1%) and performed in 17 (3.6%) cases. This highlights the importance of having alternative diagnostic options in place when CVS is not feasible or yields inconclusive results. The requirement for AC in a significant percentage of cases should prompt healthcare providers to be prepared for such scenarios and provide adequate counseling to patients. When discussing options for invasive prenatal diagnostic testing, it is essential to convey this information to each patient. Parents should be informed that AC may be necessary in the case of inconclusive results or the presence of mosaicism following CVS.^[19]^

Despite the benefits, CVS procedures carry a small risk of complications, such as miscarriage. We observed a few complications associated with CVS in our study. The procedure-related miscarriage occurred in 7 (1.4%) of pregnancies and 3 of them (0.64%) were structurally and genetically normal. It is important to note that the overall rate of complications was relatively low, with the majority of procedures being performed without any adverse events. Recent studies affirm the safety^[20]^ and effectiveness of CVS for early prenatal diagnosis, and it entails not a higher risk of procedure failure and fetal loss than amniocentesis and no interventions.^[21–23]^

The study acknowledges limitations, including its retrospective nature, and being conducted at a single center which may introduce bias and limit the generalizability of our findings. Factors such as the number of needle inserts, placental location, and operator experiences were not systematically recorded, potentially influencing outcomes.

## 5. Conclusions

This study contributes valuable insights into the indications, results, and complications of prenatal invasive diagnostic procedures, particularly CVS. Although effective, the risks and limitations of CVS should be carefully evaluated and conveyed to parents as part of the decision-making process. Continued research and advancements in prenatal genetic screening and diagnostic techniques are warranted to enhance accuracy and minimize risks. Larger sample sizes and multicenter collaborations in future studies would further strengthen the evidence.

## Author contributions

**Data curation:** Melda Kuyucu, Kadri Murat Erdogan, Duygu Adiyaman, Bahar Konuralp Atakul, Yasar Bekir Kutbay.

**Formal analysis:** Duygu Adiyaman, Bahar Konuralp Atakul, Yasar Bekir Kutbay.

**Methodology:** Melda Kuyucu.

**Supervision:** Melda Kuyucu, Hakan Golbasi, Ayse Filiz Gokmen Karasu, Mehmet Özeren.

**Visualization:** Hakan Golbasi, Ayse Filiz Gokmen Karasu, Mehmet Ozeren.

**Writing – original draft:** Melda Kuyucu.

**Writing – review & editing:** Mehmet Ozeren.
